# Focusing on *Gordonia* Infections: Distribution, Antimicrobial Susceptibilities and Phylogeny

**DOI:** 10.3390/antibiotics12111568

**Published:** 2023-10-26

**Authors:** Silvia Pino-Rosa, María J. Medina-Pascual, Gema Carrasco, Noelia Garrido, Pilar Villalón, Mónica Valiente, Sylvia Valdezate

**Affiliations:** Reference and Research Laboratory for Taxonomy, National Centre of Microbiology, Instituto de Salud Carlos III, Majadahonda, 28220 Madrid, Spain; silvia.pino@isciii.es (S.P.-R.); mjmedina@isciii.es (M.J.M.-P.); gcarrasco@isciii.es (G.C.); noegarrido@isciii.es (N.G.); pvillalon@isciii.es (P.V.); monica.valiente@isciii.es (M.V.)

**Keywords:** *Gordonia* spp. *Gordonia sputi*, *Gordonia bronchialis*, *Gordonia terrae*, *Gordonia otitidis*, *sec*A1, resistance, pneumonia, bacteremia

## Abstract

The immunosuppression conditions and the presence of medical devices in patients favor the *Gordonia* infections. However, the features of this aerobic actinomycete have been little explored. Strains (*n* = 164) were characterized with 16S rDNA and *sec*A1 genes to define their phylogenetic relationships, and subjected to broth microdilution to profile the antimicrobial susceptibilities of *Gordonia* species that caused infections in Spain during the 2005–2021 period. Four out of the eleven identified species were responsible for 86.0% of the infections: *Gordonia sputi* (53.0%), *Gordonia bronchialis* (18.3%), *Gordonia terrae* (8.5%) and *Gordonia otitidis* (6.1%). Respiratory tract infections (61.6%) and bacteremia (21.9%) were the most common infections. The *sec*A1 gene resolved the inconclusive identification, and two major clonal lineages were observed for *G. sputi* and *G. bronchialis*. Species showed a wide antimicrobial susceptibility profile. Cefoxitin resistance varies depending on the species, reaching 94.2% for *G. sputi* and 36.0% for *G. terrae*. What is noteworthy is the minocycline resistance in *G. sputi* (11.5%), the clarithromycin resistance in *G. bronchialis sec*A1 lineage II (30.0%) and the amoxicillin–clavulanate and cefepime resistance in *G. terrae* (21.4% and 42.8%, respectively). *G. sputi* and *G. bronchialis* stand out as the prevalent species causing infections in Spain. Resistance against cefoxitin and other antimicrobials should be considered.

## 1. Introduction

The genus *Gordonia* is an aerobic actinomycete that includes at the time of writing 53 validly published species (https://lpsn.dsmz.de/genus/gordonia, accessed on 28 August 2023), *Gordonia bronchialis* being the type species. It belongs to the family *Gordoniaceae* together with the genera *Williamsia* and *Jongsikchunia* in the suborder *Corynebacteriales* (https://lpsn.dsmz.de/family/gordoniaceae accessed on 25 October 2023) [1,2]. *Gordonia* encompasses a variety of ecological versatile species that have been isolated from a multitude of environments [3,4]. They are transferred with aerosols to humans, causing opportunistic infections in apparently healthy and in immunocompromised patients [5,6,7,8,9].

*G. bronchialis*, *Gordonia polyisoprenivorans*, *Gordonia sputi* and *Gordonia terrae* are described as major human pathogens [10,11,12,13]. However, other species such as *Gordonia aichiensis*, *Gordonia amicalis*, *Gordonia araiii*, *Gordonia effuse*, *Gordonia hongkongensis*, *Gordonia otitidis*, *Gordonia rubropertincta* and *Gordonia westfalica* are also responsible for human infections [11,12,13,14,15,16,17,18].

Unlike *Nocardia* spp., the most common aerobic actinomycete that received an increasing interest in the last decade [19], there is very limited information about the *Gordonia* species involved in human infections. They are rare, predominating the descriptions of catheter-related bloodstream infections, sternal wound infections and soft tissue and bone infections [9,11,15,20,21]. Patients with immunocompromised status due to congenital or acquired immunodeficiencies (AIDS infection, hypogammaglobulinemia, leukemia, solid organs cancer, transplant recipients, Hodgkin’s disease, etc.), or other underlying diseases (chronic hepatitis B virus infection, diabetes mellitus, chronic obstructive pulmonary disease, vasopressor medication, obesity), are susceptible to suffer *Gordonia* infections [5,6,11,12,13]. Also, immunocompetent patients are susceptible due to the presence of foreign bodies including central lines and shunts, being the main risk factor of this acquisition [6,17,20,22].

As for other organisms, the accurate species identification is crucial to understand the epidemiology, clinical features, treatment and outcome of *Gordonia* infections. So far, the most reliable is the sequencing of 16S rDNA [6], supported with *sec*A1 and *gyr*B genes [23,24]. However, *sec*A1 was preferred because of difficulties on amplification and sequencing of the *gyr*B gene. *Gordonia* phylogeny, based in both genes, has been mainly explored for type or reference strains [23].

The features of this aerobic actinomycete have been little explored, and because of its rarity, the infections produced with *Gordonia* species are not well understood. The aims of this work were to study the species distribution of the *Gordonia* infection in Spanish patients, and their respective antimicrobial susceptibilities. To gain insight in the knowledge of the diversity of *Gordonia* species involved in clinical infections, phylogenies based on 16S rDNA and *sec*A1 genes were also studied.

## 2. Results and Discussion

### 2.1. Distribution of Gordonia Species in Human Infections

One hundred and sixty-four strains of the genus *Gordonia* were identified in Spain during the entire period (17 years). *G. sputi* stood out as the predominant species, accounting for 53.0% (87 strains), followed by G. bronchialis (18.3%, 30 strains). Another nine species were identified: *Gordonia terrae* (8.5%, 14 strains), *G. otitidis* (6.1%, 10 strains), *G. aichiensis* (4.8%, 8 strains), *Gordonia alkanivorans*, *Gordonia araii*, *G. hongkongensis*, *Gordonia iterans*, *Gordonia jinhuaensis*, *G. polyisoprenivorans* and *Gordonia* spp. (≤2.4% for each one, ≤4 strains). According to the species distribution, four groups can be established, high prevalence (≥50%), medium prevalence (<20%), low prevalence (<10%) and other species (<5%), as is recorded in Table 1, together with the sample sources. The average and range of submission for *G. sputi* and *G. bronchialis* were 5.1 and 1–10 strains/per year, and 1.8 and 1–5 strains/per year, respectively.

The *Gordonia* strains were predominately isolated from the respiratory tract (61.6%, 101 samples) (Table 1). The remaining were collected from blood (21.9%, 36), soft tissue and bone (12.2%, 20) and other sites (five strains from urinary tract, one from central nervous system and one from bile fluid). The reported clinical manifestations of infections caused by the high and medium prevalent species, *G. sputi* and *G. bronchialis*, included primary and secondary bacteremia [11,13,15], bone infections [8], brain abscess [21], breast abscess [25], endocarditis [26,27], endophthalmitis [28], meningitis [29], peritonitis [24,30], pneumonia [11,12], soft tissue infection [14,18,20], sternal infections [9,31] and surgical site infections [32].

In our study, *G. sputi* (*n* = 2) and *G. bronchialis* (*n* = 2) were responsible for urinary tract infections (UTIs), not described previously. *G. terrae* has been described as an emerging pathogen [6], and the cause of bacteremia [6,15], cholecystitis [33], granulomatous mastitis [34], palpebral abscess [35] and peritonitis [30]. Here, *G. terrae* caused respiratory tract and soft tissue infections (it was isolated from the respiratory samples of ten patients with tuberculosis suspicion and from one wound sample). *G. otitidis* has been reported as responsible for external otitis [36] and bacteriemia [15], and here it was found as an agent of respiratory infections and UTI (seven sputum and one urine sample).

For the remaining *Gordonia* species, not-yet-described infections were observed in our study, as occurring for (i) *G. aichiensis* previously collected in blood as the cause of bacteremia [37] and here it was isolated as being responsible for respiratory tract infections in four patients; (ii) *G. araii* has been responsible for post-surgical injury and skin infection [22,38] and here it also appeared in respiratory infections; (iii) *G. hongkongensis* has been collected from blood and peritoneal dialysis effluent [16], and it was detected in two sputum and one abscess samples; (iv) *G. iterans* has been obtained from sputum as the cause of pneumonia [39], and here in bacteremia and soft tissue infection; and last, (v) *G. polysoprenivorans* has been described as the cause of bacteremia [5,12,15] and endocarditis [10], and here it was collected from two sputum and one abscess samples as responsible for respiratory and soft tissue infections. To our knowledge, there have not been reported descriptions of infections caused by *G. alkanivorans* and *G. jinhuaensis*. In our study, these species were isolated from sample of patients with soft tissue and respiratory infections, respectively.

The ability of some *Gordonia* species to attach to and colonize the surface of medical devices (central lines and shunts) with biofilm formation produces a significant number of catheter-related bloodstream or intravascular device infections [22]. This occurs for *G. polyisoprenivorans* that produces biosurfactants that help to form biofilm onto the catheter, decreasing antibiotic penetration [10]. This ability, together with the possible presence of a urinary catheter, could explain the UTIs caused by *G. sputi*, *G. bronchialis* and *G. otitidis*. However, this acquisition route was not confirmed or ruled out in our study. In this work, other species (*G. terrae*, *G. otitidis*, *G. aichiensis*, *G. hongkongensis*, *G. iterans*, *G. polyisoprenivorans* and *G. jinhuaensis*) can be the probable etiological agent of respiratory infections, due to the clinical information provided at the time of their submission. However, some of these isolations could be colonization more than true infections such as that occurring for *Nocardia* in patients with chronic pulmonary disease [19].

### 2.2. Gordonia Species Identification

Most of *Gordonia* strains reached a good identification via the 16S rDNA gene (87.2%). Twenty strains were identified at the species level via the *sec*A1 gene as *G. terrae* (14), *G. hongkongensis* (3), *G. jinhuaensis* (1), *G. otitidis* (1) and *G. polyisoprenivorans* (1) with identities ≥ 99.0% respective to their reference strain. Only one strain (CNM20140419) was not identified at the species level with 16S rDNA and *sec*A1 genes, but with neither using the *gyr*B target. Using the 16S rDNA gene, CNM20140419 showed the higher identities (98.7%) respective to *G. sputi* DSM 43896T, *G. aichiensis* DSM 43978T and *Gordonia insulae* MMS17-SY073T strains. Using the *sec*A1 gene, the best matching was reached against *G. hongkongensis* HKU50T, *G. alkanivorans* DSM 44369T and *G. bronchialis* DSM 43247T (≈90%). Finally, with the *gyr*B gene, the best identity was respective to *Gordonia namibiensis* DSM 44568, *G. bronchialis* DSM 43247T and *Gordonia rubripertincta* ATCC 14,352 (≈85.5%). Previous described cut-offs for *sec*A1 and for *gyr*B genes (≥93.5%) were considered [39]. This strain could be a new species or not formally described yet.

MALDI-TOFF did not show any identification in nearly half of the studied strains (*n* = 65), as occurring for *G. sputi* (10 not identified strains/28 tested strains), *G. bronchialis* (9/12), *G. terrae* (1/4), *G. otitidis* (2/2), *G. aichiensis* (3/3), *G. hongkongensis* (1/3, two misidentified as G. terrae), *G. iterans* (2/2), *G. polyisoprenivorans* (4/4), *G. alkanivorans* (1/1), *G. arai* (2/2), *G. jinhuaensis* (1/1) and *Gordonia* sp. (1/1). These pitfalls could be partially due to the lack of information regarding many *Gordonia* species in the MALDI-TOFF databases [16,27,40], being necessary to increase the number of spectra for each species and to add other species (i.e., *G. otitidis*).

### 2.3. Antimicrobial Susceptibilities of Gordonia Species

In previous works, the data about the antimicrobial susceptibility profiles of *Gordonia* species were reduced to the results of the strain that produce the clinical case, or a low number of strains, or just a review of the literature. Fortunately, *Gordonia* spp. are usually described as antibiotic-sensitive [6,20,23,30,41] as it is shown in Table 2 for the 164 strains of the 11 species identified as *Gordonia* spp. Their wide susceptibility spectrum includes β-lactams and carbapenems; amikacin and tobramycin; clarithromycin; doxycycline and minocycline; ciprofloxacin and moxifloxacin; trimethoprim and sulphametoxazole; and linezolid.

However, the results showed some details: (i) the lack of activity of cefoxitin against *Gordonia* strains, with overall resistance rates of 76.6%, and this cefoxitin resistance affects all species studied, and it ranged from 35.7% for *G. terrae* to 94.2% for *G. sputi*, and (ii) some species harbored a differential resistance profile, as occurring for *G. sputi* against minocycline (11.5%), *G. bronchialis* respective to clarithromycin (30.0%) and *G. terrae* against amoxicillin–clavulanate and cefepime (21.4% and 42.8%, respectively). Regarding tigecycline, when the corresponding EUCAST susceptibility breakpoint criteria of ≤0.5 mg/L for *Staphylococcus* spp. and Enterobacterales organisms was used, a high resistance rate was found for *G. bronchialis* (40%), and for *G. sputi* and *G. terrae* (≈30% for each one) [42].

*Gordonia* spp. degrade xenobiotics, environmental pollutants and biodegradable natural polymers, and transform or synthesize possibly useful compounds for environmental and industrial biotechnology [43,44]. These abilities could orientate toward thinking that *Gordonia* should be intrinsically an antimicrobial multiresistant bacteria. A further note is that susceptible profiles of the different *Gordonia* species were mainly observed in our study. This feature differs greatly from those described for the *Nocardia* species [45,46]. Both organisms proceed from environmental sites (mainly soil) and they are also subjected to the effect of bioactive compounds as antimicrobial products synthesized using actinobacteria in this niche including *Streptomyces* spp., among others [47]. In general, *Nocardia* strains show an expanded resistance profile, which affects different classes of antibiotics, and with specific drug pattern types according to the *Nocardia* species [48]. At this moment, these events do not occur for *Gordonia* spp.

There is not a standardized treatment for *Gordonia* infections. But due to their large susceptible phenotype, the patients are successfully treated with a variety of antimicrobial regimens, usually for several weeks or months [12,23,27]. *Gordonia* infections can be difficult to identify and treat [30,33], being recommendable to undergo an accurate identification and antimicrobial susceptibility testing for tailoring the treatment, and to remove the medical device [22].

### 2.4. Phylogenetic Analysis of Gordonia spp.

#### 2.4.1. 16S rDNA and *sec*A1 phylogeny

Two major clusters were observed in the phylogenetic tree based on 16S rDNA sequences (adjusted to 943-pb, Figure S1): *G. sputi*, *G. aichiensis*, *G. otitidis*, *G. jinhuaensis* and *G. polyisoprenivorans* grouped together in one cluster, and *G. alkanivorans* and *G. bronchialis* in a second cluster. Meanwhile, *G. araii* and *G. iterans*, and *G. terrae* and *G. hongkongensis*, were placed in minor third and fourth clusters. The bootstrap values were low (≤50), except for the *G. terrae* and *G. hongkongensis* cluster (96). In contrast, the values for branches that grouped strains belonging to the same species were high (average of 90), except for the branches of *G. aichiensis* and three *G. otitidis* strains (63 and 47, respectively). In another study [23], similar clusters were observed for 23 *Gordonia* species, except for *G. alkanivorans* (it was grouped together with *G. terrae*).

**Figure 1:**
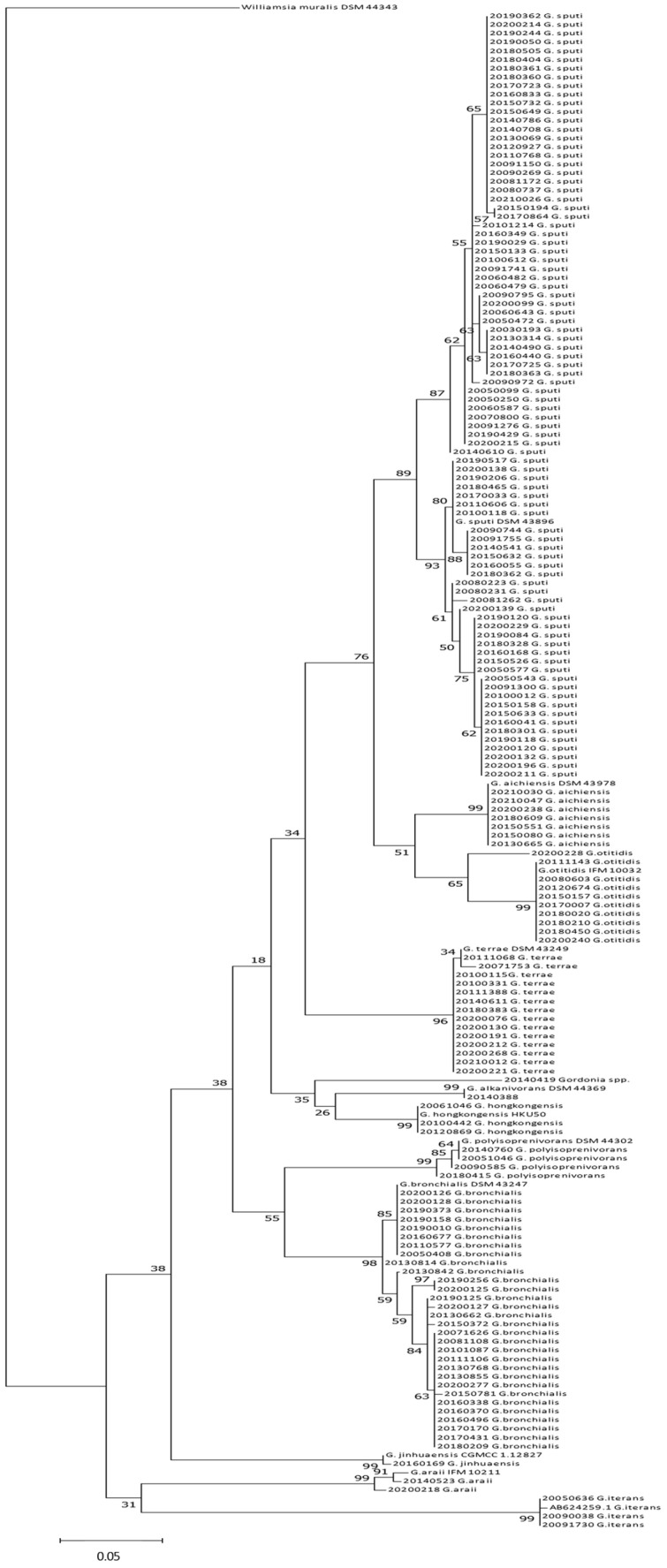
*sec*A1 sequence-based (337 bp alignment positions) phylogenetic tree of clinical *Gordonia* strains with those of reference strains (*n* = 175 strains), and *Williamsia muralis* reference strain MA140/96 as outgroup using MEGA7 [49]. The evolutionary history was inferred by using the Maximum Likelihood method based on the Tamura 3-parameter model with 1000 replications for bootstrap values. The optimal tree is shown. The tree is drawn to scale, with branch lengths in the same units as those of the evolutionary distances used to infer the phylogenetic tree. A discrete Gamma distribution was used to model evolutionary rate differences among sites (5 categories (+G, parameter = 0.5004)).

Through the *sec*A1 gene, some 16S rDNA clustering remained similar for *G. sputi*, *G. aichiensis* and *G. otitidis*, but not for *G. polyisoprenivorans* that grouped together with *G. bronchialis*, and for *G. alkanivorans* that grouped together with *G. hongkongensis* (Figure 1). This different position of *G. polyisoprenivorans* was also previously seen [23]. Three independent branches were seen for *G. jinhuaensis*, *G. araii* and *G. iterans*. *G. terrae* and *G. hongkongensis* gathered in the same branch with the 16S rDNA gene (with no differences in a studied fragment of 1.166 bp), but they were separated with the *sec*A1 gene, whereas *G. terrae* appeared in a unique cluster, and *G. hongkongensis* did with *G. alkanivorans*. The strain CNM20140419 *Gordonia* spp. was placed between the two branches for the 16S rDNA gene of *G. polyisoprenivorans* and *G. jinhuaensis*. Meanwhile, in the tree of the *sec*A1 gene, this strain placed in a cluster was together with *G. alkanivorans* and *G. hongkongensis.*

The partial *sec*A1 gene provided significant base diversity among all strains (HGDI = 0.954), and also for *G. sputi* and *G. bronchialis* groups (0.89 and 0.75, respectively), but it was very low for *G. terrae* and *G. otitidis* groups (Table 3). For the *G. sputi* group, the identity or sequence similarity respective to the type strain ranged from 100.0% to 95.6%; for *G. bronchialis*, from 100.0% to 88.7%; for *G. terrae*, from 100.0% to 99.6%; and for *G. otitidis*, from 100.0% to 96.0%. Low values of identity for *sec*A1 showed the great diversity of this gene for a single species. This could be due to the presence of different clonal lineages of intra-species that are onwards seen. In a previous work, Kang et al. reported a range of secA1 identity values from 82 to 98% for different species [23].

#### 2.4.2. *Gordonia* Species with High Prevalence: *G. sputi*

**Figure 2:**
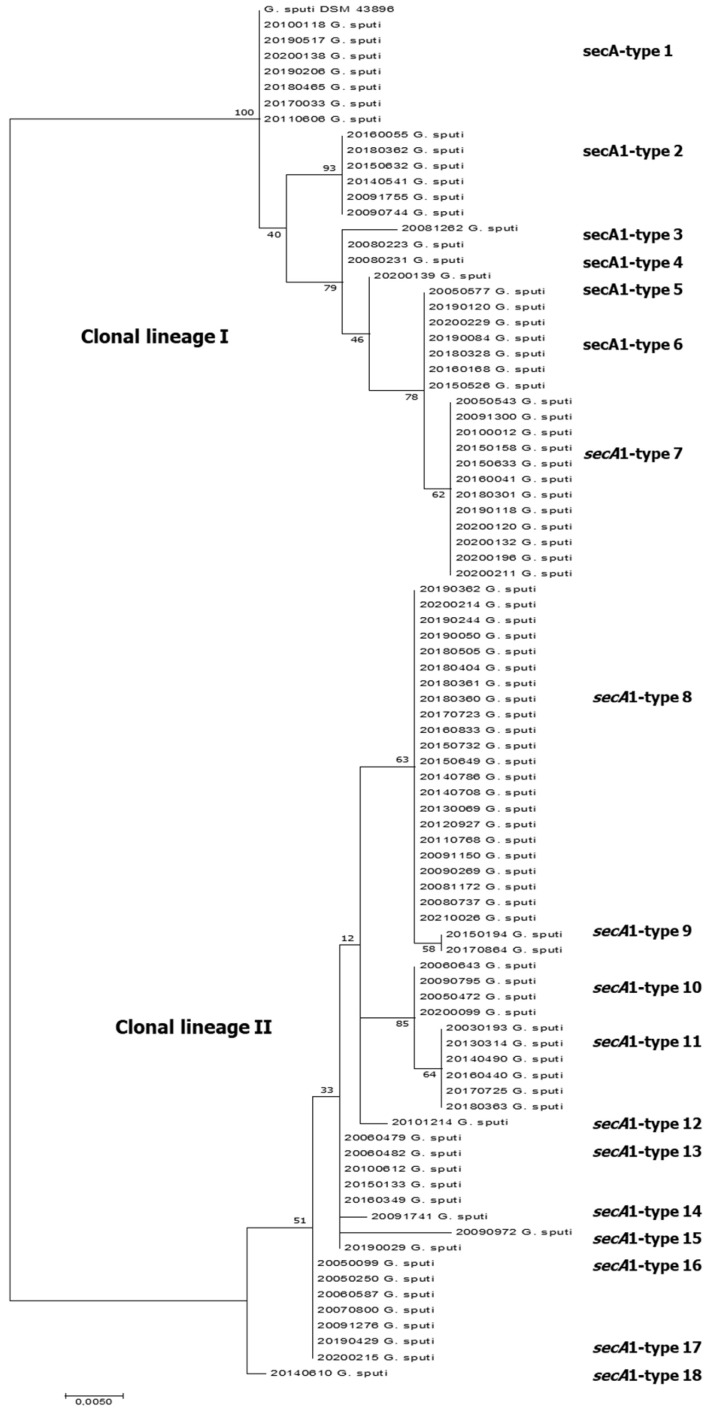
*sec*A1 sequence-based phylogenetic tree of clinical *G. sputi* strains with reference *G. sputi* DSM 43,896 (*n* = 88 strains, 453 bp alignment positions) using MEGA7 [49]. The evolutionary history was inferred by using the Maximum Likelihood method based on the Tamura 3-parameter model with 1000 replications for bootstrap values. The optimal tree is shown. The tree is drawn to scale, with branch lengths in the same units as those of the evolutionary distances used to infer the phylogenetic tree. A discrete Gamma distribution was used to model evolutionary rate differences among sites (5 categories (+G, parameter = 0.0500)).

Six different 16S rDNA haplotypes were observed in the *G. sputi* population (*n*= 87 strains). The 16S-haplotype of the *G. sputi* DSM 43896T strain was identical for 81 strains. Six single nucleotide polymorphisms (SNPs) (positions: 322, 375, 549, 817, 997 and 1099) appeared in one strain for each one, and one SNP (position 1228) was shared by two strains. Thirty-five variable sites were detected in the *sec*A1 partial sequences (454 bp) of all the strains, resulting in 18 different *sec*A1-haplotypes (Table 3). Sixteen SNPs were detected in more than half of the studied population (positions 441, 442, 459, 501, 507, 531, 615, 672, 678, 689, 759, 792, 804, 825, 852 and 861 respective to the complete *sec*A1 gene of *G. sputi* ATCC 29,627 GenBank accession no. JAAXP0010000001). Five amino acid changes were identified: Glu-148 → Gln, in 51 strains; Pro-271 → Ser, in 12 strains (added to 148-Gln change); Glu-266 → Asp, in 6 strains; and Val-152 → Ile plus Ala-155 → Asp, in one strain. The corresponding phylogenetic tree (Figure 2) shows two clonal lineages, clonal lineage I (represented by the strain *G. sputi* DSM 43896T) and II. Clonal lineage I was constituted by 36 strains distributed in seven *sec*A1-types (coded as A1–A7), whereas clonal lineage II comprises 51 strains and ten *sec*A1-types (A8–A18). The *sec*A1-types A7 and A8 (with 12/36 and 22/51 strains, respectively) were predominant for each lineage. 

The nucleotide identity for all *G. sputi* strains (≤95.6% for ≤20 SNPs/454 bp) increased for clonal lineage I respective to *G. sputi* DSM 43896T (≤98.4% for ≤7 SNPs), while lower identity remained for lineage II respective to *sec*A1-type A8 (≤95.6% for ≤20 SNPs, respectively). Therefore, lineage II was more diverse than lineage I. Until 2014, the involvement of clonal lineage II in the human infections of the Spanish patients was double than lineage I, but since 2015, the clinical implications of both lineages were similar. These lineages were not seen so clear in the *sec*A1 gene phylogenies for *G. sputi* strains using *G. bronchialis* DSM 43247 as the outgroup (Figure S2).

#### 2.4.3. *Gordonia* Species with Medium Prevalence: *G. bronchialis*

**Figure 3:**
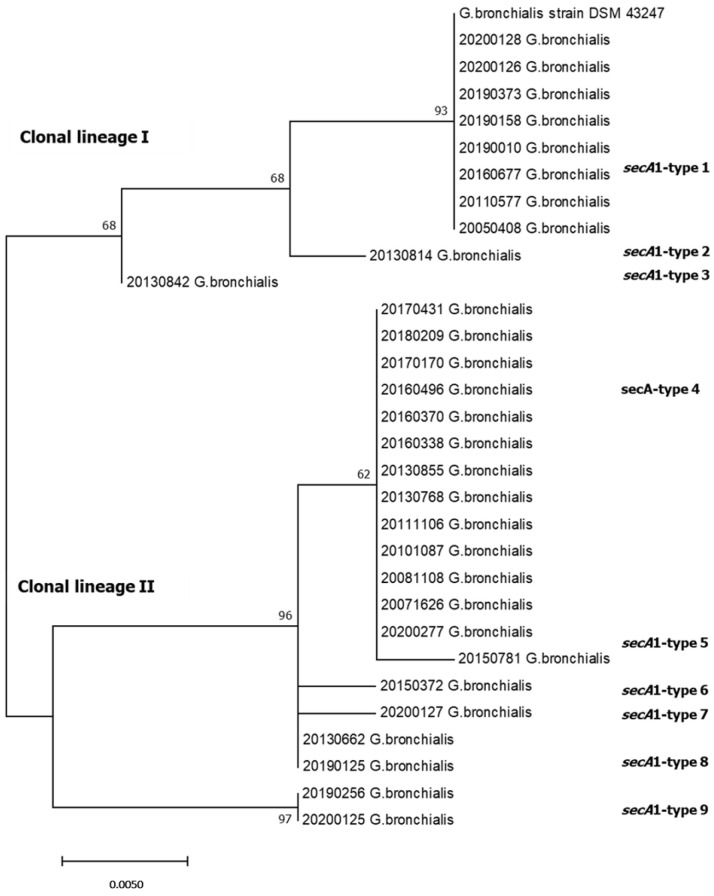
*sec*A1 sequence-based phylogenetic tree of clinical *G. bronchialis* strains with reference *G. bronchialis* DSM 43247 (*n* = 31 strains, 335 bp alignment positions) using MEGA7 [49]. The evolutionary history was inferred by using the Maximum Likelihood method based on the Tamura 3-parameter model with 1000 replications for bootstrap values. The optimal tree is shown. The tree is drawn to scale, with branch lengths in the same units as those of the evolutionary distances used to infer the phylogenetic tree. A discrete Gamma distribution was used to model evolutionary rate differences among sites (5 categories (+G, parameter = 0.0500)).

The 16S-haplotype of the reference strain was found in all *G. bronchialis strains* (*n* = 30). This is unlike the *sec*A1 partial sequences, which showed 15 segregating sites respective to the *G. bronchialis* DSM 43247 strain, resulting in nine *sec*A1-haplotypes (Table 3). Nine of these SNPs were very prevalent (≥46%) (positions 480, 507, 540, 543, 651, 672, 690, 738 and 801 respective to the complete *sec*A1 gene of *G. sputi* ATCC 29,627 GenBank accession no. JAAXP0010000001). Three amino acid replacements were produced, Asp-180 → Glu, Glu-231 → Gln and Arg-258 → Gln, in 14, 21 and 2 strains, respectively. Three clonal lineages could be observed in the phylogenetic tree (Figure 3), created with the same suggested model mentioned above: clonal lineage I, represented by the strain *G. bronchialis* DSM 43247T with *sec*A1-type A1 (eight strains), and types A2–A3 (one strain for each one); clonal lineage II, with the predominant *sec*A1-type A4 (thirteen strains), and types A5–A8 (one strain for each one); and clonal lineage III, represented by type A9 (two strains). In the studied period, clonal lineage II produced double the clinical cases than other lineages. It should be highlighted that seven out of nine clarithromycin-resistant strains belonged to clonal lineage II (*n* = 18).

Two outbreaks of sternal wound infections with *G. bronchialis*, where nurse scrub was identified as the vector for contamination of the surgical wounds, were described [50,51]. To investigate the clonal relation among strains collected in four patients hospitalized in the same ward during 6 months with previous cardiac surgery, *sec*A1 typing was applied. Three *sec*A1-types were detected (A1, A7 and A9). To distinguish two strains with *sec*A1-type A1, the *gyr*B gene sequencing was carried out, resulting in two different alleles, discarding the outbreak scenario.

#### 2.4.4. *Gordonia* Species with Low Prevalence: *G. terrae* and *G. otitidis*

In the case of *G. terrae*, a common haplotype of 16S rDNA was shared by the reference DSM 43249T strain and the studied strains. Three *sec*A1-haplotypes were seen: one silent SNP in 12 strains (identity of 99.8%); one silent SNP and the mutation Ile-177 → Val in one strain (identity of 99.6%); and the *sec*A1-type of the type strain, in one strain. Regarding *G. otitidis*, seven strains showed the same 16S haplotype compared to the reference strain, and three strains showed one change (position 433). A unique *sec*A1-haplotype was observed for all strains, except one strain that showed 18 silent SNPs respective to the reference strain (identity of 96%). These strains with changes in 16S or *sec*A1 genes did not show any difference in the clinical or susceptibility features.

#### 2.4.5. Other *Gordonia* species

Regarding the other seven detected species, differences respective to the 16S rDNA haplotype of the corresponding reference strain were found for *G. alkanivorans* (position 704, one strain), *G. iterans* (position 403, three strains) and *G. jinhuaensis* (position 1357, one strain). Meanwhile, identical 16S haplotypes of the reference strain were found for strains belonging to *G. aichiensis* (eight strains), *G. araii* (two strains) and *G. polyisoprenivorans* (four strains) species. It should be noted that 16S rDNA sequences of three *G. hongkongensis* strains showed a full identity respective to the reference *G. hongkongensis* strain HKU50, but also respective to *G. terrae* NRRL B-16283 (GenBank accession nos. NR_152022.1 and CP029604.1, respectively). Differences in a partial sequence of the *sec*A1 gene were detected for two *G. araii* strains (identities of 99.1–98.1%, 4–8 silent SNPs/432 bp), for three strains of *G. iterans* (99.8%, 1 SNP/432 bp, Met-94 → Ileu), for one strain of *G. jinhuaensis* (99.8%, 1 silent SNP/506 bp) and for three out of four *G. polyisoprenivorans* strains (99.7–98.6%, 1–6 silent SNPs/443 bp), respective to the corresponding reference strains. The same *sec*A1-haplotype compared to the corresponding reference strain was found for all strains of *G. aichiensis*, *G. alkanivorans* and *G. hongkongensis*. In addition, when the *sec*A1 of *G. hongkongensis* strain HKU50 was compared to *G. terrae* DSM 43249T, the identity was 94.31% (24 SNPs/422 bp), allowing the correct species assignment.

In the adaptation and survival of bacteria, including manipulating host cells and competing for resources with other microorganisms, the protein secretion plays many roles in bacteria [52]. This happens in the cell envelope biogenesis and maintenance, and in the delivery of adherence and pathogenic effector proteins to the cell surface [53]. The Sec translocase is responsible for the translocation of unfolded proteins across membranes. The two components of the Sec translocase are the cytoplasmic motor protein, SecA1, and the membrane-embedded channel, SecYEG. During protein secretion, SecYEG engages with the cytosolic motor ATPase SecA1, and together they pass pre-proteins with a short N-terminal cleavable signal sequence across the membrane, whilst still having an unfolded conformation [53,54]. The vital role and variability of SecA1 in bacteria becomes connected to the *sec*A1 gene as a strong candidate to perform identification and phylogeny. So, it had been previously described for *Gordonia* species [23], as for other related genera such as a *Mycobacterium* and *Nocardia* [55,56]. To obtain an accurate identification of *Gordonia* strains, different approaches have been used, in special genome-based approaches [57]. The analysis of the *sec*A1 gene can provide a quick and simple panoramic view of the diversity of the populations of *Gordonia* species (as it has been seen for *G. sputi* and *G. bronchialis*) with clinical and resistance implications.

## 3. Materials and Methods

### 3.1. Strains and Target Genes for Identification

Strains were submitted from different Spanish hospitals to the Reference and Research Laboratory for Taxonomy of National Center for Microbiology (CNM, Majadahonda, Madrid, Spain) for species identification. The strains were isolated from patients with signs and symptoms of bacterial infection in 31 provinces of 13 Spanish Autonomous Communities between 2005 and 2021 (17 years). Only one strain per patient was considered in this study. Strains were grown on Columbia agar supplemented with 5% (*v*/*v*) sheep blood and buffered charcoal–yeast extract agar (BCYE) for 48–72 h at 37 °C under aerobic conditions. DNA was extracted with the boiling method and amplifications were performed using Ready-To-Go PCR Beads (Amersham Biosciences, Buchinghamshire, UK) with the following targets to identification: (i) a full 16S rDNA gene sequence analysis, with primers fD1 and rP2 for amplification [58], and E781 and U1115 for sequencing [59]; (ii) the *sec*A1 gene with primers SecA1-F and SecA1-R [23]; and (iii) the *gyr*B gene with primers UP-1 and UP-2 for no identified strains [23] (Table S1). Specific primers for the gyrB sequence of G. bronchialis 123F and 1248Rev were designed to study the possible relation of G. bronchialis strains. The amplification products were electrophoresed and purified using an ExoSAP-IT reagent (GE Healthcare, Livingston, NJ, USA) and sequenced with capillary electrophoresis in an ABI Prism 3100 apparatus (Applied Biosystems, Foster City, CA, USA).

### 3.2. Species Assignment

The sequences of the 16S rDNA and *sec*A1 genes were compared against those corresponding to Gordonia-type strains (https://lpsn.dsmz.de/genus/gordonia; accessed on 8 August 2023) using the BLAST algorithm v.2.2.10 (http://www.ncbi.nlm.nih.gov/BLAST, accessed on 8 August 2023) [32]. Similarity values of ≥99.6% for the 16S rDNA gene, following CLSI MM18 guidelines (CLSI, 2008) [60], were deemed to indicate the same species. 

### 3.3. Antimicrobial Susceptibility Testing

Susceptibilities to first-line recommended drugs (amoxicilin–clavulanate, cefoxitin, ceftriaxone, imipenem, tobramycin, amikacin, clarithromycin, minocycline, doxycycline, ciprofloxacin, moxifloxacin, linezolid and trimethoprim–sulfamethoxazole) and to cefepime and tigecycline were determined with the broth microdilution method using Sensititre^®^ RAPMYCO microtiter plates (ThermoFisher, Inc., Cleveland, OH, USA), according to CLSI M24-A2 guidelines for aerobic actinomycetes using the *Staphylococcus aureus* ATCC 29,213 control strain [61]. Minimum inhibitory concentration (MIC) was determined after 48 h of incubation at 37 °C with the evaluation of the growth control well (if needed, incubation was extended 24 h more). Resistance was recorded according to the CLSI criteria [62]. The tigecycline susceptibility breakpoint for *Staphylococcus* spp. and *Enterobacterales* (≤0.5 mg/L) was used for tentative interpretation [42]. Intermediate values were categorized as resistant.

### 3.4. 16S rDNA and secA1 Phylogeny

Sequences were assembled using SEQ-Man software (DNASTAR, Inc., Madison, WI, USA) and adjusted for a phylogenetic analysis to coincide with the length of the shortest sequence using BioEdit software [63]. The Hunter–Gaston discrimination index (HGDI) [64] and summary statistics for the analysis of multi-locus population genetics were assessed using DNA Sequence Polymorphism (DnaSP) software [65].

A phylogenetic assessment of all collected populations of Gordonia strains was undertaken with the 16S rDNA gene and *sec*A1 genes. For the species with high and medium prevalence, dendograms based on the *sec*A1 gene were created. Phylogenetic trees were constructed using maximum-likelihood methods [66], with bootstrap analyses based on 1000 resamplings. Branches corresponding to partitions that were reproduced in <50% of bootstrap replicates were collapsed. The evolutionary distance between the nucleotide and amino acid sequences of the *sec*A1 gene was determined using the Tamura 3-parameter mode with gamma distribution (five categories and by assuming that a certain fraction of sites is evolutionary invariable), as it is suggested as the best DNA model with MEGA7 [49]. *Williamsia muralis*-type strain MA140/96 (GenBank accession no. NR_037083.1) was included as an outgroup for the phylogeny of the 16S rDNA and *sec*A1 genes.

Sixty-five strains that belonged to different species were analyzed with a matrix-assisted laser desorption ionization time of flight MS (MALDI-TOF MS)-based system (Vitek MS, SARAMIS premium software, bioMerieux, Madrid, Spain), following the procedure recommended by the manufacturer. Briefly, target slides were inoculated into the spots by picking a freshly grown overnight colony and overlaid with a matrix solution (1 µL of a-cyano-4-hydroxycinnamic acid) using a complete protocol of protein extraction with formic acid and acetonitrile.

## 4. Conclusions

The two main species responsible for the *Gordonia* infections in Spain are *G. sputi* and *G. bronchialis* (71.3%). Species assignation was correctly performed in 87.2% of the clinical *Gordonia* strains with the 16S rDNA gene. The *sec*A1 gene resolves the inconclusive identification, giving a finer species distinction among closely related species (*G. terrae* and *G. hongkongensis*), discriminates among clinical strains and defines phylogenetic relationships at the inter- and intra-species level. In this way, the *sec*A1 gene provides a panoramic view of their diversity with clinical and resistance implications. To our knowledge, this is the first study that explores susceptibilities and the phylogenies of a wide population of *Gordonia* clinical strains to better understand the increasing clinical importance of the infections caused by this genus.

## Figures and Tables

**Table 1:** Distribution of the *Gordonia* species in clinical infections in Spain according to sample source in the 2005–2021 period.

	No. of Strains (%) ^b^							
Species (No. and %) ^a^	Blood	CNS ^c^	Respiratory Tract			Soft Tissue/Bone	Urine	Other
			Sputum	Bronchial Fluid	Lung/Pleural Fluid			
**High prevalence**								
*G. sputi* (87, 53.0)	18 (20.7%)	1 (1.1%)	60 (69.0%)	1(1.1%)	1 (1.1%)	4 (6.0%)	2 (2.3%)	0
**Medium prevalence**								
*G. bronchialis* (30, 18.3)	6 (20.0%)	0	13 (43.3%)	0	0	9 (30.0%)	2 (6.6%)	0
**Low prevalence**								
*G. terrae* (14, 8.5)	2 (14.3)	0	6 (42.8)	3 (21.4)	1 (7.1)	2 (14.3)	0	0
*G. otitidis* (10, 6.1%)	2 (20.0)	0	7 (70.0)	0	0	0	1 (10.0)	0
**Other species**								
*G. aichiensis* (8, 4.8)	3	0	4	0	0	0	0	1
*G. alkanivorans* (1, 0.6)	0	0	0	0	0	1	0	0
*G. araii* (2, 1.2)	0	0	0	1	0	1	0	0
*G. hongkongensis* (3, 1.8%)	3	0	0	0	0	0	0	0
*G. iterans* (3, 1.8)	1	0	1	0	0	1	0	0
*G. jinhuaensis* (1, 0.6)	0	0	1	0	0	0	0	0
*G. polyisoprenivorans* (4, 2.4%)	1	0	2	0	0	1	0	0
*Gordonia* spp. (1, 0.6)	0	0	0	0	0	1	0	0
**Total** (164, 100) ^a^	36 (21.9)	1 (0.61)	94 (57.3)	5 (3.0)	2 (1.2)	20 (12.2)	5 (3.0)	1 (0.6)

^a^ Figures in parentheses refer to the number of strains and percentage for the cited species; ^b^ percentage respective to the number of strains for a given species; ^c^ CNS, central nervous system.

**Table 2:** Antimicrobial susceptibilities of the *Gordonia* species responsible for clinical infections in Spain over the 17-year study period.

Species	*Gordonia* *sputi*	*Gordonia* *bronchialis*	*Gordonia* *terrae*	*Gordonia* *otitidis*	*Gordonia* *aichiensis*	*Gordonia* *hongkongensis*	*Gordonia* *iterans*	*Gordonia* *polyisoprenivorans*	*Other Species* ^b^	*All Species*
(No. of Strains, % ^a^)	(87, 53.0%)	(30, 18.3%)	(14, 8.5%)	(10, 6.1%)	(8, 4.8%)	(3, 1.8%)	(3, 1.8%)	(4, 2.4%)	(5, 3.0%)	(164, 100%)
**Amoxycillin–clavulanate ^c,d^**										
Range	≤2	≤2	≤2–32	≤2	≤2	≤2–8	≤2	≤2	≤2	≤2–32
MIC50/MIC90 ^e^	≤2/≤2	≤2/≤2	4/16	≤2/≤2	≤2/≤2	4/8	≤2/≤2	≤2/≤2	≤2/≤2	2/2
Resistance ^f,g^	0 (0.0)	0 (0.0)	3 (21.4)	0 (0.0)	0	0	0	0	0	3 (1.8)
**Ceftriaxone**										
Range	≤4	≤4	≤4–128	≤4	≤4	≤4	≤4	≤4	≤4	≤4–128
MIC50/MIC90	≤4/≤4	≤4/≤4	≤4/≤4	≤4/≤4	≤4/≤4	≤4/≤4	≤4/≤4	≤4/≤4	≤4/≤4	≤4/≤4
Resistance	0 (0.0)	0 (0.0)	1 (0.78)	0 (0.0)	0	0	0	0	0	1 (0.6)
**Cefoxitin**										
Range	8–32	≤4–32	≤4–32	≤4–16	16–16	≤4–32	≤4–8	8–16	≤4–32	≤4–32
MIC50/MIC90	16/32	16/16	8/16	8/16	16/16	16/32	8/8	16/16	16/16	32/32
Resistance ^h^	82 (94.2)	20 (66.7)	5 (35.7)	5 (50.0)	8	2	0	3	3	128 (76.6)
**Cefepime**										
Range	≤1	≤1	≤1–>32	≤1	≤1	≤1–32	≤1	≤1	≤1	≤1–>32
MIC50/MIC90	≤1/≤1	≤1/≤1	≤1/>32	≤1/≤1	≤1/≤1	≤1/32	≤1/≤1	≤1/≤1	≤1/≤1	≤1/≤1
Resistance	0 (0.0)	0 (0.0)	6 (42.8)	0 (0.0)	0	1	0	0	0	7 (4.3)
**Imipenem**										
Range	≤2	≤2	≤2	≤2	≤2	≤2	≤2	≤2	≤2	≤2
MIC50/MIC90	≤2/≤2	≤2/≤2	≤2/≤2	≤2/≤2	≤2/≤2	≤2/≤2	≤2/≤2	≤2/≤2	≤2/≤2	≤2/≤2
Resistance	0 (0.0)	0 (0.0)	0 (0.0)	0 (0.0)	0	0	0	0	0	0 (0.0)
**Amikacin**										
Range	≤1	≤1	≤1	≤1	≤1	≤1	≤1	≤1	≤1	≤1
MIC50/MIC90	≤1/≤1	≤1/≤1	≤1/≤1	≤1/≤1	≤1/≤1	≤1/≤1	≤1/≤1	≤1/≤1	≤1/≤1	≤1/≤1
Resistance	0 (0.0)	0 (0.0)	0 (0.0)	0 (0.0)	0	0	0	0	0	0
**Tobramycin**										
Range	≤1	≤1	≤1	≤1	≤1	≤1	≤1	≤1	≤1–4	≤1–4
MIC50/MIC90	≤1/≤1	≤1/≤1	≤1/≤1	≤1/≤1	≤1/≤1	≤1/≤1	≤1/≤1	≤1/≤1	≤1/2	≤1/2
Resistance	0 (0.0)	0 (0.0)	0 (0.0)	0 (0.0)	0	0	0	0	0	0 (0.0)
**Clarithromycin**										
Range	≤0.06–2	≤0.06–8	0.12–4	≤0.06–0.25	0.12–0.25	≤0.06–2	2	≤0.06	≤0.06–4	≤0.06–8
MIC50/MIC90	≤0.06/0,12	2/4	2/2	0.12/0.25	0.12/0.25	≤0.06/2	2/2	≤0.06/≤0.06	≤0.5/1	1/2
Resistance	0 (0.0)	9 (30)	1 (7.1)	0 (0.0)	0	0	0	0	1	9 (5.5)
**Doxycycline**										
Range	0.25–4	0.25–1	≤0.12–0.5	0.25–0.5	≤0.12–1	0.25–0.5	0.25	0.25–0.5	≤0.12–0.5	≤0.12–4
MIC50/MIC90	0.5/1	0.5/1	0.25/0.5	0.25/0.5	0.5/0.5	0.25/0.5	0.25/0.25	0.25/0.5	0.25/0.5	0.5/1
Resistance	1 (1.1)	0 (0.0)	0 (0.0)	0 (0.0)	0	0	0	0	0	1 (0.6)
**Minocycline**										
Range	≤1–2	≤1	≤1	≤1	≤1	≤1	≤1	≤1	≤1	≤1
MIC50/MIC90	≤1/2	≤1/≤1	≤1/≤1	≤1/≤1	≤1/≤1	≤1/≤1	≤1/≤1	≤1/≤1	≤1/≤1	≤1/≤1
Resistance	10 (11.5)	0 (0.0)	0 (0.0)	0 (0.0)	0	0	0	0	0	10 (6.1)
**Ciprofloxacin**										
Range	≤0.12–2	≤0.12–1	≤0.12	≤0.12	≤0.12	≤0.12	≤0.12	≤0.12	≤0.12–0.25	≤0.12–2
MIC50/MIC90	≤0.12/≤0.12	≤0.12/≤0.12	≤0.12/≤0.12	≤0.12/≤0.12	≤0.12/≤0.12	≤0.12/≤0.12	≤0.12/≤0.12	≤0.12/≤0.12	≤0.12/≤0.12	≤0.12/≤0.12
Resistance	0 (0.0)	0 (0.0)	0 (0.0)	0 (0.0)	0	0	0	0	0	0 (0.0)
**Moxifloxacin**										
Range	≤0.25	≤0.25–0.5	≤0.25	≤0.25	≤0.25	≤0.25	≤0.25	≤0.25	≤0.25	≤0.25–0.5
MIC50/MIC90	≤0.25/≤0.25	≤0.25/≤0.25	≤0.25/≤0.25	≤0.25/≤0.25	≤0.25/≤0.25	≤0.25/≤0.25	≤0.25/≤0.25	≤0.25/≤0.25	≤0.25/≤0.25	≤0.25/≤0.25
Resistance	0 (0.0)	0 (0.0)	0 (0.0)	0 (0.0)	0	0	0	0	0	0 (0.0)
**Trimethoprim–sulphamethoxazole ^d^**										
Range	≤0.25–0.5	≤0.25	≤0.25–0.5	≤0.25–0.5	≤0.25–0.5	≤0.25–1	≤0.25	≤0.25	≤0.25–0.5	≤0.25–1
MIC50/MIC90	≤0.25/≤0.25	≤0.25/≤0.25	≤0.25/≤0.25	≤0.25/≤0.25	≤0.25/≤0.25	1/1	≤0.25/≤0.25	≤0.25/≤0.25	≤0.25/≤0.25	≤0.25/≤0.25
Resistance	0 (0.0)	0 (0.0)	0 (0.0)	0 (0.0)	0	0	0	0	0	0 (0.0)
**Tigecycline**										
Range	0.25–2	0.12–1	0.25–1	0.25–0.5	0.12->4	0.25–0.5	0.25	0.25–1	0.25–1	0.12–4
MIC50/MIC90	0.5/1	0.5/1	0.5/1	0.5/0.5	0.5/1	0.25/0.5	0.25/0.25	0.5/1	0.5/1	0.5/1
Resistance ^i^	25 (28.7)	12 (40.0)	4 (28.5)	0 (0.0)	2	0	0	1	1	45 (43.0%)
**Linezolid**										
Range	≤1–2	≤1	≤1	≤1	≤1	≤1	≤1	≤1	≤1	≤1
MIC50/MIC90	≤1/≤1	≤1/≤1	≤1/≤1	≤1/≤1	≤1/≤1	≤1/≤1	≤1/≤1	≤1/≤1	≤1/≤1	≤1/≤1
Resistance	0 (0.0)	0 (0.0)	0 (0.0)	0 (0.0)	0	0	0	0	0	0 (0.0)

^a^ The percentage represented with respect to the total number of identified *Gordonia* strains (*n* = 164). ^b^ Other *Gordonia* species included one *G. alkanivorans*, two *G. araii*, 1 *G. jinhuaensis* and one *Gordonia* spp. strains. ^c^ The range of antimicrobial dilutions (mg/L) in the RAPMYCO panel is the following: amoxycillin–clavulanate, 2/1–64/32; ceftriaxone, 4–64; cefoxitin, 4–128; cefepime, 1–32; imipenem, 2–64; amikacin, 1–64; tobramycin, 1–16; clarithromycin, 0.06–16; doxycycline, 0.12–16; minocycline, 1–8; ciprofloxacin, 0.12–4; moxifloxacin, 0.25–8; trimethoprim–sulphamethoxazole, 0.25/4.75–8/152; tigecycline, 0.015–4; and linezolid, 1–32. ^d^ Concentrations of amoxicillin–clavulanic-acid (ratio of 2:1) and trimethoprim–sulphamethoxazole (ratio of 1:19) are expressed in terms of amoxicillin and trimethoprim, respectively. ^e^ In total, 50% and 90% MICs at which 50% and 90% of the strains were inhibited, respectively. ^f^ Number and percentage of resistant strains and intermediate-resistance strains for this species. ^g^ In total, 2011 CLSI interpretative criteria for broth microdilutions were taken into account for defining non-susceptibility (mg/L): amoxycillin–clavulanate ≥ 16/8; ceftriaxone ≥ 16; cefepime ≥ 16; imipenem ≥ 8; amikacin ≥ 16; tobramycin ≥ 8; clarithromycin ≥ 4; doxycycline ≥ 2; minocycline ≥ 2; ciprofloxacin ≥ 2; moxifloxacin ≥ 2; trimethoprim–sulphamethoxazole ≥ 4/76; and linezolid ≥16 (CLSI, 2011). ^h^ For cefoxitin, the ceftriaxone and cefepime breakpoint of ≥16 mg/L was used. ^i^ For tigecycline, the corresponding susceptibility breakpoint of ≤0.5 mg/L for *Staphylococcus* spp. and *Enterobacterales* following EUCAST criteria was used.

**Table 3:** Summary statistics for the analysis of 16S rRNA and *sec*A1 population genetics of the high, medium and low prevalent species of *Gordonia* isolated from clinical samples in Spain between 2005 and 2021.

Species (No. of Strains)	Genes (bp) ^a^	Haplotype Number (HGDI, S^2^, SD) ^b^	SNP Number (Nucleotide Diversity) ^c^	SNPs Per Strain (Average, Mode) ^d^
*Gordonia* spp. (*n =* 174) ^e^	16S rRNA (954)	20 (0.737, 0.00085, 0.029)	81 (≤1.37)	--
	*sec*A (337)	44 (0.954, 0.00003, 0.006)	165 (≤8.53)	--
**High prevalence**				
*G. sputi* (*n* = 87)	16S rRNA (1088)	6 (0.132, 0.0024, 0.049)	7 (0.017)	0–2 (0.18, 0)
	*sec*A (454)	18 (0.890, 0.00033, 0.018)	35 (2.55)	0–20 (7, 20)
Clonal lineage I (*n* = 36) ^f^	*sec*A (454)	7 (0.803, 0.00107, 0.033)	10 (0.864)	0–7 (4.4, 7)
Clonal lineage II (*n* = 51) ^f^	*sec*A (454)	11 (0.773, 0.00231, 0.048)	15 (0.567)	0–20 (2, 0) ^g^
**Medium prevalence**				
*G. bronchialis* (*n* = 30)	16S rRNA (1093)	1	0	0–1 (0.0, 0)
	*sec*A (336)	9 (0.751, 0.00342, 0.059)	15 (1.5)	0–11 (6.7, 10)
Clonal lineage I (*n* = 10) ^h^	*sec*A (336)	3 (0.345, 0.02967, 0.172)	5 (0.358)	0–4 (0.7, 0)
Clonal lineage II (*n* = 20) ^h^	*sec*A (336)	6 (0.579, 0.0142, 0.124)	10 (0.559)	0–10 (3.15, 0)
**Low prevalence**				
*G. terrae* (*n* = 14)	16S rRNA (1167)	2 (0.0, 0.0, 0.0)	1 (0.0)	0
	*sec*A (462)	3 (0.362, 0.02098, 0.145)	3 (0.132)	0–2 (0.14, 1)
*G. otitidis* (*n* = 10)	16S rRNA (1293)	2 (0.436, 0.0177, 0.133)	1 (0.034)	0–1 (0, 0)
	*sec*A (451)	2 (0.182, 0.0261, 0.144)	18 (5.2)	0–18 (1.6, 0)

^a^ Analyzed size in number of base pars; ^b^ HGDI, S2 and SD correspond to the Hunter and Gaston discrimination index, the variance and standard deviation, respectively; ^c^ the nucleotide diversity (defined as the average number of nucleotide differences per site between two sequences) is expressed as a percentage among strains of each group (Nei and Kumar, 2000); ^d^ respective to reference strain of each species; ^e^ this analysis includes 164 clinical strains and 10 reference strains; ^f^ 6 SNPs were identified respective to *G. sputi* DMS 43,896 strain (*sec*A1-type) and respective to the prevalent *sec*A-type (A8-type) for clonal lineages I and II, respectively; ^g^ data of clonal lineage II respective to *G. sputi* DMS 43,896 strain were 16–20 (18.4, 19); ^h^ SNPs were identified respective to *G. bronchialis* DMS 43247 strain (A1-type) and respective to prevalent *sec*A-type (A4-type) for clonal lineage I and II, respectively.

## Data Availability

The new 16S rDNA and *sec*A1 sequences were deposited in GenBank under the accession numbers OK030649–OK030656 and OK030657–OK030691, respectively.

## Supplementary Materials

The following supporting information can be downloaded at: https://www.mdpi.com/article/10.3390/antibiotics12111568/s1, Figure S1. 16S rDNA sequence-based (943 bp alignment positions) phylogenetic tree of clinical *Gordonia* strains with those of reference strains and *Williamsia muralis* reference strain MA140/96 as outgroup (*n* = 175 strains) using MEGA7 [49]. The evolutionary history was inferred by using the Maximum Likelihood method based on the Tamura 3-parameter model with 1000 replications for bootstrap values. The optimal tree is shown. The tree is drawn to scale, with branch lengths in the same units as those of the evolutionary distances used to infer the phylogenetic tree. A discrete Gamma distribution was used to model evolutionary rate differences among sites (5 categories (+G, parameter = 0.1000)); Figure S2. *sec*A1 sequence-based phylogenetic tree of clinical *G. sputi* strains with *G. sputi* DSM 43896, and *G. bronchialis* DSM 43247 as outgroup in Figure S2a (*n* = 89 strains, 453 bp alignment positions). *sec*A1 sequence-based phylogenetic tree of clinical *G. bronchialis* strains with *G. bronchialis* DSM 43247, and *G. sputi* DSM 43896 as outgroup in Figure S2b (*n* = 32 strains, 335 bp alignment positions), using MEGA7 [49]. The evolutionary history was inferred by using the Maximum Likelihood method based on the Tamura 3-parameter mode with 1000 replications for bootstrap values. The optimal tree is shown. The tree is drawn to scale, with branch lengths in the same units as those of the evolutionary distances used to infer the phylogenetic tree. A discrete Gamma distribution was used to model evolutionary rate differences among sites (5 categories (+G, parameter = 2.8492 and 0.1977)); Table S1. Primers and conditions of amplification used for *Gordonia* species.

## References

[1] Tsukamura M. (1971). Proposal of a new genus, *Gordona*, for slightly acid-fast organisms occurring in sputa of patients with pulmonary disease and in soil. J. Gen. Microbiol..

[2] Parte A.C., Sardà Carbasse J., Meier-Kolthoff J.P., Reimer L.C., Göker M. (2020). List of Prokaryotic names with Standing in Nomenclature (LPSN) moves to the DSMZ. Int. J. Syst. Evol. Microbiol..

[3] Andalibi F., Fatahi-Bafghi M. (2017). *Gordonia*: Isolation and identification in clinical samples and role in biotechnology. Fol. Microbiol..

[4] Sowani H., Kulkarni M., Zinjarde S. (2018). An insight into the ecology, diversity and adaptations of *Gordonia* species. Critical Rev. Microbiol..

[5] Kempf V.A., Schmalzing M., Yassin A.F., Schaal K.P., Baumeister D., Arenskötter M., Steinbüchel A., Autenrieth I.B. (2004). *Gordonia polyisoprenivorans* septicemia in a bone marrow transplant patient. Eur. J. Clin. Microbiol. Infect. Dis..

[6] Blaschke A.J., Bender J., Byington C.L., Korgenski K., Daly J., Petti C.A., Pavia A.T., Ampofo K. (2007). *Gordonia* species: Emerging pathogens in pediatric patients that are identified by 16S ribosomal RNA gene sequencing. Clin. Infect. Dis..

[7] Lai C.C., Wang C.Y., Liu C.Y., Tan C.K., Lin S.H., Liao C.H., Chou C.H., Huang Y.T., Lin H.I., Hsueh P.R. (2010). Infections caused by *Gordonia* species at a medical centre in Taiwan, 1997 to 2008. Clin. Microbiol. Infect..

[8] Siddiqui N., Toumeh A., Georgescu C. (2012). Tibial osteomyelitis caused by *Gordonia bronchialis* in an immunocompetent patient. J. Clin. Microbiol..

[9] Ambesh P., Kapoor A., Kazmi D.H., Elsheshtawy M., Shetty V., Lin Y.S., Kamholz S. (2019). Sternal osteomyelitis by *Gordonia bronchialis* in an immunocompetent patient after open heart surgery. Ann. Card. Anaesth..

[10] Verma P., Brown J.M., Nunez V.H., Morey R.E., Steigerwalt A.G., Pellegrini G.J., Kessler H.A. (2006). Native valve endocarditis due to *Gordonia polyisoprenivorans*: Case report and review of literature of bloodstream infections caused by *Gordonia* species. J. Clin. Microbiol..

[11] Johnson J.A., Onderdonk A.B., Cosimi L.A., Yawetz S., Lasker B.A., Bolcen S.J., Brown J.M., Marty F. (2011). *Gordonia bronchialis* bacteremia and pleural infection: Case report and review of the literature. J. Clin. Microbiol..

[12] Ding X., Yu Y., Chen M., Wang C., Kang Y., Li H., Lou J. (2017). Bacteremia due to *Gordonia polyisoprenivorans*: Case report and review of literature. BMC Infect. Dis..

[13] Guerrero C., Casañe C., Antequera P., Candel C., Blázquez R. (2014). Catheter-related bloodstream infection caused by *Gordonia terrae* in a bone-marrow transplant patient: Case report and review of the literature. J. Med. Microbiol. Case Rep..

[14] Lai C.C., Hsieh J.H., Tsai H.Y., Liao C.H., Hsueh P.R. (2012). Cutaneous Infection Caused by *Gordonia amicalis* after a Traumatic Injury. J. Clin. Microbiol..

[15] Ramanan R., Deziel P.J., Wengenack N.L. (2012). *Gordonia* Bacteremia. J. Clin. Microbiol..

[16] Tsang C.C., Xiong L., Poon R.W.S., Chen J.H.K., Leung K.W., Lam J.Y.W., Wu A.K.L., Chan J.F.W., Lau S.K.P., Woo P.C.Y. (2016). *Gordonia hongkongensis* sp. nov., isolated from blood culture and peritoneal dialysis effluent of patients in Hong Kong. Int. J. Syst. Evol. Microbiol..

[17] Akrami K., Coletta J., Mehta S., Fierer J. (2017). *Gordonia* sternal wound infection treated with ceftaroline: Case report and literature review. J. Med. Microbiol. Case Rep..

[18] Gueneau R., Blanchet D., Rodriguez-Nava V., Bergeron E., Soulier M., Bestandji N., Demar M., Couppie P., Blaizot R. (2020). Actinomycetoma caused by *Gordonia westfalica*: First reported case of human infection. New Microbes New Infect..

[19] Margalit I., Lebeaux D., Tishler O., Goldberg E., Bishara J., Yahav D., Coussement J. (2021). How do I manage nocardiosis?. Clin. Microbiol. Infect..

[20] Bartolomé-Álvarez J., Sáez-Nieto J.A., Escudero-Jiménez A., Barba-Rodríguez N., Galán-Ros J., Carrasco G., Muñoz-Izquierdo M.P. (2016). Cutaneous abscess due to *Gordonia bronchialis*: Case report and literature review. Rev. Esp. Quimioter..

[21] Eribi A., Al-Amri K., Al-Jabri A., Osman A., Mohamed Elfadil O. (2020). *Gordonia sputi* related multiple brain abscesses, an AIDS-presenting illness: Thinking outside the box. IDCases.

[22] Jannat-Khah D.P., Halsey E.S., Lasker B.A., Steigerwalt A.G., Hinrikson H.P., Brown J.M. (2009). *Gordonia araii* infection associated with an orthopedic device and review of the literature on medical device-associated *Gordonia* infections. J. Clin. Microbiol..

[23] Kang Y., Takeda K., Yazawa K., Mikami Y. (2009). Phylogenetic studies of *Gordonia* species based on *gyr*B and *sec*A1 gene analyses. Mycopathologia.

[24] Lam J.Y., Wu A.K., Leung W.S., Cheung I., Tsang C.C., Chen J.H., Chan J.F., Tse C.W., Lee R.A., Lau S.K. (2015). *Gordonia* species as emerging causes of continuous-ambulatory-peritoneal-dialysis-related peritonitis identified by 16S rDNA and secA1 gene sequencing and matrix-assisted laser desorption ionization-time of flight mass spectrometry (MALDI-TOF MS). J. Clin. Microbiol..

[25] Werno A.M., Anderson T.P., Chambers S.T., Laird H.M., Murdoch D.R. (2005). Recurrent breast abscess caused by *Gordonia bronchialis* in an immunocompetent patient. J. Clin. Microbiol..

[26] Lesens O., Hansmann Y., Riegel P., Heller R., Benaissa-Djellouli M., Martinot M., Petit H., Christmann D. (2000). Bacteremia and endocarditis caused by a *Gordonia* species in a patient with a central venous catheter. Emerg. Infect. Dis..

[27] Mormeneo Bayo S., Palacián Ruíz M.P., Asin Samper U., Millán Lou M.I., Pascual Catalán A., Villuendas Usón M.C. (2022). Pacemaker-induced endocarditis by *Gordonia bronchialis*. Enferm. Infecc. Microbiol. Clin. (Engl. Ed.).

[28] Choi R., Strnad L., Flaxel C.J., Lauer A.K., Suhler E.B. (2019). *Gordonia bronchialis*—Associated Endophthalmitis, Oregon, USA. Emerg. Infect. Dis..

[29] Martín D., Barrios A., Domingo D., Sánchez P., Sánchez M., Ruiz-Dassy A., Miqueleiz A., Sanz J. (2017). Cerebrospinal fluid shunt-associated meningitis caused by *Gordonia sputi*: Case report and review of the literature. Infez. Med..

[30] Hou C., Yang Y., Li Z. (2017). A Chinese patient with peritoneal dialysis-related peritonitis caused by *Gordonia terrae*: A case report. BMC Infect. Dis..

[31] Chang J.H., Ji M., Hong H.L., Choi S.H., Kim Y.S., Chung C.H., Sung H., Kim M.N. (2014). Sternal Osteomyelitis Caused by *Gordonia bronchialis* after Open-Heart Surgery. Infect. Chemother..

[32] Drancourt M., Bollet C., Carlioz A., Martelin R., Gayral J.P., Raoult D. (2000). 16S ribosomal DNA sequence analysis of a large collection of environmental and clinical unidentifiable bacterial isolates. J. Clin. Microbiol..

[33] Gil-Sande E., Brun-Otero M., Campo-Cerecedo F., Esteban E., Aguilar L., García-de-Lomas J. (2006). Etiological misidentification by routine biochemical tests of bacteremia caused by *Gordonia terrae* infection in the course of an episode of acute cholecystitis. J. Clin. Microbiol..

[34] Zardawi I.M., Jones F., Clark D.A., Holland J. (2004). *Gordonia terrae*-induced suppurative granulomatous mastitis following nipple piercing. Pathology.

[35] Blanc V., Dalle M., Markarian A., Debunne M.V., Duplay E., Rodriguez-Nava V., Boiron P. (2007). *Gordonia terrae*: A difficult-to-diagnose emerging pathogen?. J. Clin. Microbiol..

[36] Iida S., Taniguchi H., Kageyama A., Yazawa K., Chibana H., Murata S., Nomura F., Kroppenstedt R.M., Mikami Y. (2005). *Gordonia otitidis* sp. nov., isolated from a patient with external otitis. Int. J. Syst. Evol. Microbiol..

[37] Thomas E., Lejeune F., Caillon J., Wiertlewski S., Crémet L. (2017). Premier cas clinique de bactériémie à *Gordonia aichiensis* [First case report of Gordonia aichiensis bacteremia]. Med. Mal. Infect..

[38] Muñoz-Peña C., Ocaña-Cano M.J., Amores-Antequera C., Muñoz-Peña C., Ocaña-Cano M.J., Amores-Antequera C., Cantudo-Muñoz P. (2016). Infección cutánea por *Gordonia araii* [Skin infection due to *Gordonia araii*]. Enferm. Infecc. Microbiol. Clin..

[39] Kang Y.Q., Ming H., Gonoi T., Chen Y., Cao Y., Wang Y.Y., Cheng J., Koga T., Mikami Y., Li W.J. (2014). *Gordonia iterans* sp. nov., isolated from a patient with pneumonia. Int. J. Syst. Evol. Microbiol..

[40] Ercibengoa Arana M., Alonso M., Idigoras P., Vicente D., Marimón J.M. (2018). Matrix-assisted laser desorption ionization-time of flight mass spectrometry (MALDI-TOF) score algorithm for identification of *Gordonia* species. AMB Express.

[41] Moser B.D., Pellegrini G.J., Lasker B.A., Brown J.M. (2012). Pattern of antimicrobial susceptibility obtained from blood isolates of a rare but emerging human pathogen, *Gordonia polyisoprenivorans*. Antimicrob. Agents Chemother..

[42] European Committee on Antimicrobial Susceptibility Testing (2021). Breakpoint Tables for Interpretation of MICs and Zone Diameters. Version 11.0. http://www.eucast.org.

[43] Arenskötter M., Bröker D., Steinbüchel A. (2014). Biology of the metabolically diverse genus *Gordonia*. Appl. Environ. Microbiol..

[44] Drzyzga O. (2012). The strengths and weaknesses of *Gordonia*: A review of an emerging genus with increasing biotechnological potential. Crit. Rev. Microbiol..

[45] Schlaberg R., Fisher M.A., Hanson K.E. (2014). Susceptibility profiles of *Nocardia* isolates based on current taxonomy. Antimicrob. Agents Chemother..

[46] Valdezate S., Garrido N., Carrasco G., Medina-Pascual M.J., Villalón P., Navarro A.M., Saéz-Nieto J.A. (2017). Epidemiology and susceptibility to antimicrobial agents of the main *Nocardia* species in Spain. J. Antimicrob. Chemother..

[47] Barka E.A., Vatsa P., Sanchez L., Gaveau-Vaillant N., Jacquard C., Meier-Kolthoff J.P., Klenk H.P., Clément C., Ouhdouch Y., van Wezel G.P. (2015). Taxonomy, Physiology, and Natural Products of Actinobacteria. Microbiol. Mol. Biol. Rev..

[48] Conville P.S., Brown-Elliott B.A., Smith T., Zelazny A.M. (2017). The Complexities of *Nocardia* Taxonomy and Identification. J. Clin. Microbiol..

[49] Kumar S., Stecher G., Tamura K. (2016). MEGA7, Molecular Evolutionary Genetics Analysis Version 7.0 for Bigger Datasets. Mol. Biol. Evol..

[50] Richet H.M., Craven P.C., Brown J.M., Lasker B.A., Cox C.D., McNeil M.M., Tice A.D., Jarvis W.R., Tablan O.C. (1991). A cluster of *Rhodococcus* (*Gordona*) *bronchialis* sternal-wound infections after coronary-artery bypass surgery. N. Engl. J. Med..

[51] Wright S.N., Gerry J.S., Busowski M.T., Klochko A.Y., McNulty S.G., Brown S.A., Sieger B.E., Ken Michaels P., Wallace M.R. (2012). *Gordonia bronchialis* sternal wound infection in 3 patients following open heart surgery: Intraoperative transmission from a healthcare worker. Infect. Control Hosp. Epidemiol..

[52] Green E.R., Mecsas J. (2016). Bacterial Secretion Systems: An Overview. Microbiol. Spectr..

[53] Ahdash Z., Pyle E., Allen W.J., Corey R.A., Collinson I., Politis A. (2019). HDX-MS reveals nucleotide-dependent, anti-correlated opening and closure of SecA and SecY channels of the bacterial translocon. eLife.

[54] Chen W., Komives E.A. (2021). Open, engage, bind, translocate: The multi-level dynamics of bacterial protein translocation. Structure.

[55] Zelazny A.M., Calhoun L.B., Li L., Shea Y.R. (2005). Identification of *Mycobacterium* species by *sec*A1 sequences. J. Clin. Microbiol..

[56] Conville P.S., Zelazny A.M., Witebsky F.G. (2006). Analysis of *sec*A1 gene sequences for identification of *Nocardia* species. J. Clin. Microbiol..

[57] Frantsuzova E., Bogun A., Vetrova A., Delegan Y. (2022). Methods of Identifying *Gordonia* Strains in Clinical Samples. Pathogens.

[58] Weisburg W.G., Barns S.M., Pelletier D.A., Lane D.J. (1991). 16S ribosomal DNA amplification for phylogenetic study. J. Bacteriol..

[59] Baker G.C., Smith J.J., Cowan D.A. (2003). Review and re-analysis of domain-specific 16S primers. J. Microbiol. Meth..

[60] Clinical and Laboratory Standards Institute (2008). Interpretive Criteria for Identification of Bacteria and Fungi by DNA Target Sequencing: Approved Guideline MM18-A.

[61] Clinical Laboratory Standards Institute (2018). Susceptibility Testing of Mycobacteria, Nocardia spp. and other Aerobic Actinomycetes.

[62] Clinical Laboratory Standards Institute (2011). Susceptibility Testing of Mycobacteria, Nocardiae, and Other Aerobic Actinomycetes. Approved Standard-M24-A2.

[63] Hall T.A. (1999). BioEdit: A user-friendly biological sequence alignment editor and analysis program for Windows 95/98/NT. Nucleic Acids Symp Ser..

[64] Hunter P.R., Gaston M.A. (1988). Numerical index of the discriminatory ability of typing systems: An application of Simpson’s index of diversity. J. Clin. Microbiol..

[65] Rozas J., Ferrer-Mata A., Sánchez-Del Barrio J.C., Guirao-Rico S., Librado P., Ramos-Onsins S.E., Sánchez-Gracia A. (2017). DnaSP 6, DNA Sequence Polymorphism Analysis of Large Data Sets. Mol. Biol. Evol..

[66] Felsenstein J. (1981). Evolutionary trees from DNA sequences: A maximum likelihood approach. J. Mol. Evol..

