# Supplementary material for: Focusing on Gordonia Infections: Distribution, Antimicrobial Susceptibilities and Phylogeny
Source: Antibiotics (Basel). 2023 Oct 26;12(11):1568. doi: 10.3390/antibiotics12111568 (PMC10668661; doi:10.3390/antibiotics12111568)
Supplement: Supplementary file 1 [file antibiotics-12-01568-s001.zip › 10 FigS2.pptx]

## Slide 1
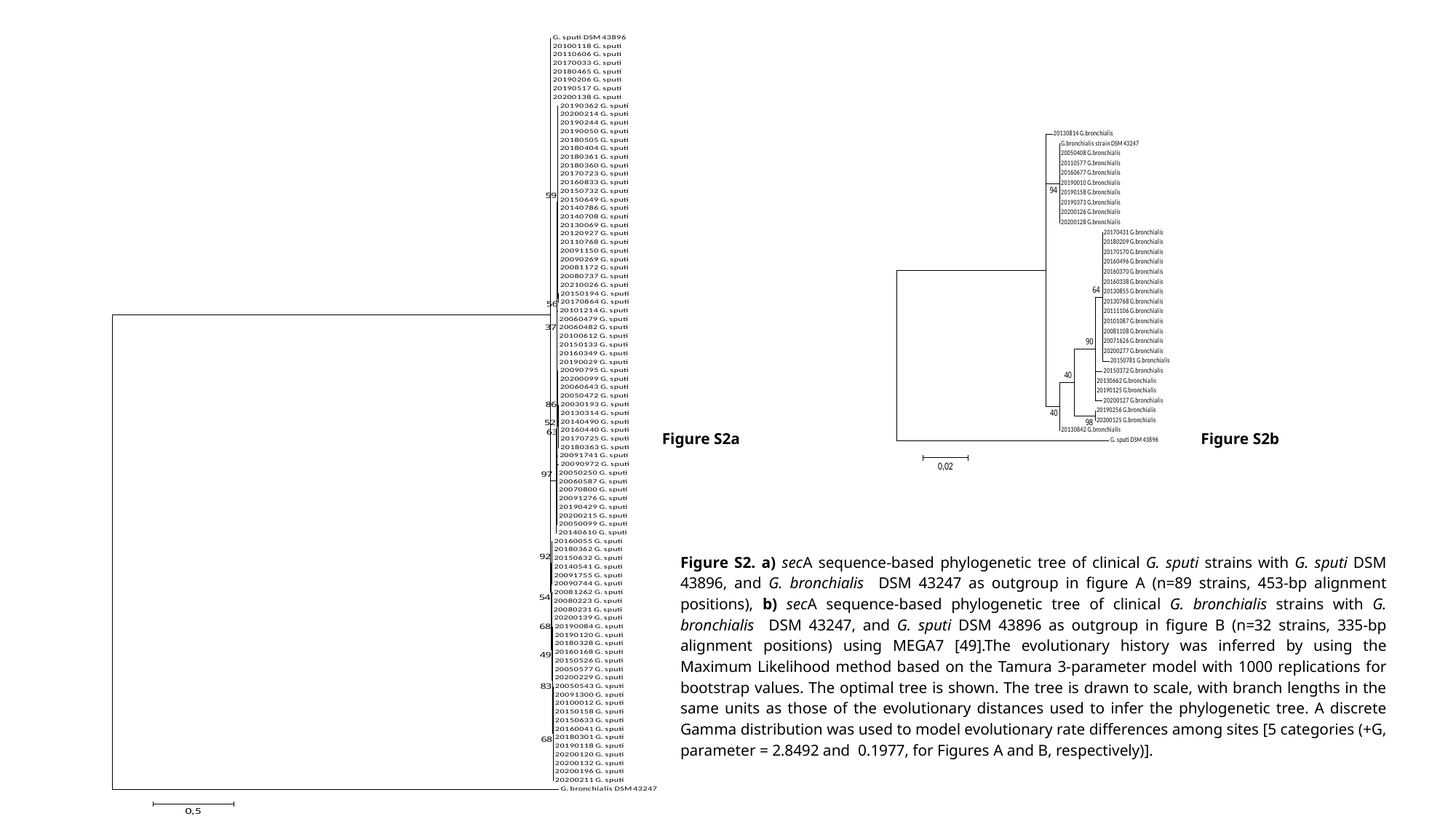

Figure S2a
Figure S2b
Figure S2. a) secA sequence-based phylogenetic tree of clinical G. sputi strains with G. sputi DSM 43896, and G. bronchialis DSM 43247 as outgroup in figure A (n=89 strains, 453-bp alignment positions), b) secA sequence-based phylogenetic tree of clinical G. bronchialis strains with G. bronchialis DSM 43247, and G. sputi DSM 43896 as outgroup in figure B (n=32 strains, 335-bp alignment positions) using MEGA7 [49].The evolutionary history was inferred by using the Maximum Likelihood method based on the Tamura 3-parameter model with 1000 replications for bootstrap values. The optimal tree is shown. The tree is drawn to scale, with branch lengths in the same units as those of the evolutionary distances used to infer the phylogenetic tree. A discrete Gamma distribution was used to model evolutionary rate differences among sites [5 categories (+G, parameter = 2.8492 and 0.1977, for Figures A and B, respectively)].
