# Supplementary material for: Focusing on Gordonia Infections: Distribution, Antimicrobial Susceptibilities and Phylogeny
Source: Antibiotics (Basel). 2023 Oct 26;12(11):1568. doi: 10.3390/antibiotics12111568 (PMC10668661; doi:10.3390/antibiotics12111568)
Supplement: Supplementary file 1 [file antibiotics-12-01568-s001.zip › 11 Table S1.pdf]

Table S1. Primers and conditions of amplification used for *Gordonia* species.

Gene	Sequence *(5´-3´)*	Size (bp)	Reference
16S rDNA amplification		1,459	[57]
fD1: forward primer	AGAGTTTGATCCTGGCTCAG		
rP2: reverse primer	ACGGCTACCTTGTTACGACTT		
16S rDNA sequencing			[58]
fD1 and rP2			
E781: forward primer	GATTAGATACCCTGGTAG		
U1115: reverse primer	GGGTTGCGCTCGTTG		
*sec*A1 amplification and sequencing		496	[23]
SecA1-F: forward primer	GTAAAACGACGGCCAGGACAGYGAGTGGATGGGYCGSGTGCACCG		
SecA1-R: reverse primer	CAGGAAACAGCTATGACGCGGACGATGTAGTCCTTGTC´		
*gyr*B amplification and sequencing		1,230	[23]
UP-1: forward primer	GAGGTCGTCATGACCCAGCTGCAYGCNGGNGGNAARTTYGA		
UP-2: reverse primer	*AGCAGCGTCGAGATGTGCTGGCCRTCNACRGCNGCRTCNGTCA*		
*Gordonia bronchialis gyr*B amplification and sequencing		1,125	This study
123F: forward primer	GAGGTCGTCATGACCCAGCTGCAYGCNGGNGGNAARTTYGA		
1248Rev: reverse primer	*AGCAGCGTCGAGATGTGCTGGCCRTCNACRGCNGCRTCNGTCA*		

The amplification reaction was performed under the following conditions: initial denaturation at 94°C for 5 min followed by 35 cycles of denaturation at 94°C for 30 s, primer annealing at 55°C for 30 s, and primer extension at 72°C for 1 min with final extension at 72°C for 10 min. The temperature of annealing of 55°C, was diminished to obtain amplification band (50°C, 46°C).
