# Supplementary material for: Focusing on Gordonia Infections: Distribution, Antimicrobial Susceptibilities and Phylogeny
Source: Antibiotics (Basel). 2023 Oct 26;12(11):1568. doi: 10.3390/antibiotics12111568 (PMC10668661; doi:10.3390/antibiotics12111568)
Supplement: Supplementary file 1 [file antibiotics-12-01568-s001.zip › 9 FigS1.pptx]

## Slide 1
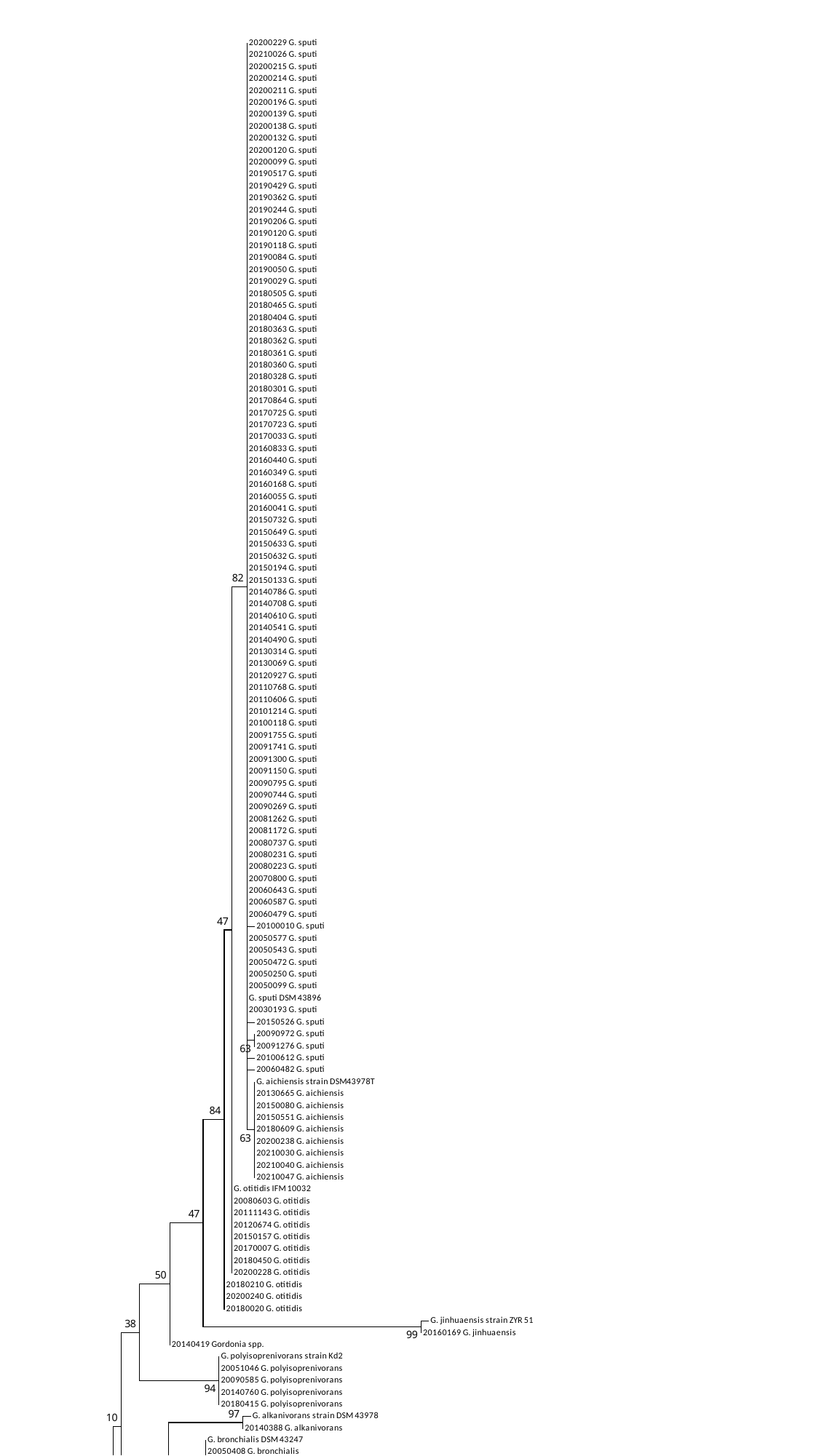

## Slide 2
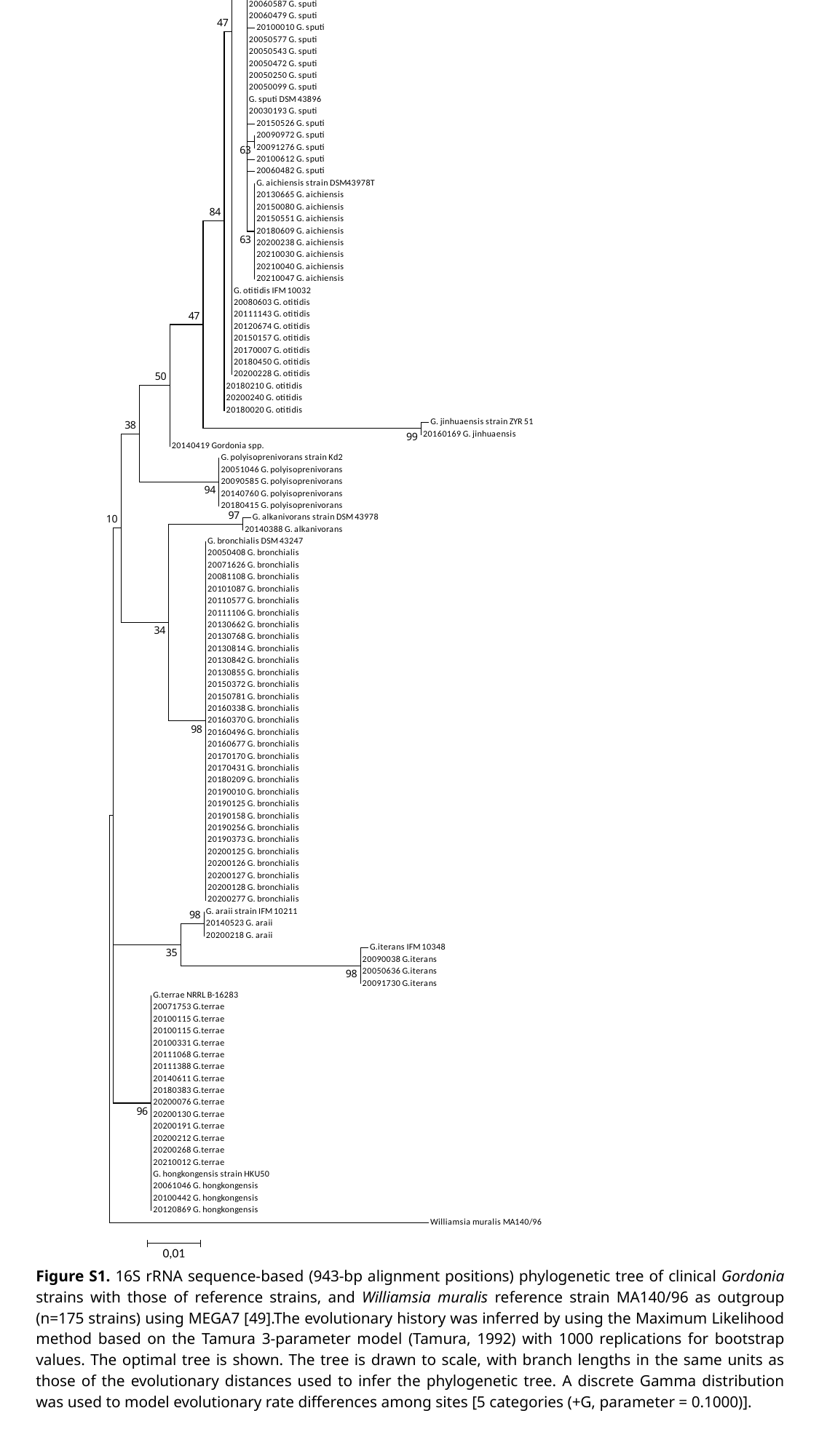

Figure S1. 16S rRNA sequence-based (943-bp alignment positions) phylogenetic tree of clinical Gordonia strains with those of reference strains, and Williamsia muralis reference strain MA140/96 as outgroup (n=175 strains) using MEGA7 [49].The evolutionary history was inferred by using the Maximum Likelihood method based on the Tamura 3-parameter model (Tamura, 1992) with 1000 replications for bootstrap values. The optimal tree is shown. The tree is drawn to scale, with branch lengths in the same units as those of the evolutionary distances used to infer the phylogenetic tree. A discrete Gamma distribution was used to model evolutionary rate differences among sites [5 categories (+G, parameter = 0.1000)].
